# Effect of high and low roughage total mixed ration diets on rumen metabolites and enzymatic profiles in crossbred cattle and buffaloes

**DOI:** 10.14202/vetworld.2017.616-622

**Published:** 2017-06-09

**Authors:** S. K. Sinha, V. B. Chaturvedi, Putan Singh, L. C. Chaudhary, Mayukh Ghosh, Swati Shivani

**Affiliations:** 1 Division of Animal Nutrition, ICAR-Indian Veterinary Research Institute, Izatnagar, Bareilly - 243 122, Uttar Pradesh, India; 2 Department of Animal Nutrition, Ranchi Veterinary College, Birsa Agricultural University, Kanke, Ranchi - 834 006, Jharkhand, India; 3 Department of Veterinary Biochemistry, Ranchi Veterinary College, Birsa Agricultural University, Kanke, Ranchi - 834 006, Jharkhand, India; 4 Division of Animal Nutrition, ICAR-National Dairy Research Institute, Karnal - 132 001, Haryana, India

**Keywords:** buffalo, crossbred cattle, rumen ecology, rumen metabolites

## Abstract

**Aim::**

A comparative study was conducted on crossbred cattle and buffaloes to investigate the effect of feeding high and low roughage total mixed ration (TMR) diets on rumen metabolites and enzymatic profiles.

**Materials and Methods::**

Three rumen-fistulated crossbred cattle and buffalo were randomly assigned as per 3×3 switch over design for 21-days. Three TMR diets consisting of concentrate mixture, wheat straw and green maize fodder in the ratios of (T_1_) 60:20:20, (T_2_) 40:30:30, and (T_3_) 20:40:40, respectively, were fed to the animals *ad libitum*. Rumen liquor samples were collected at 0, 2, 4, 6, and 8 h post feeding for the estimation of rumen biochemical parameters on 2 consecutive days in each trial.

**Results::**

The lactic acid concentration and pH value were comparable in both species and treatments. Feed intake (99.77±2.51 g/kg body weight), ruminal ammonia nitrogen, and total nitrogen were significantly (p<0.05) higher in buffalo and in treatment group fed with high concentrate diet. Production of total volatile fatty acids (VFAs) was non-significant (p>0.05) among treatments and significantly (p<0.05) greater in crossbred cattle than buffaloes. Molar proportions of individual VFAs propionate (C3), propionate:butyrate (C3:C4), and (acetate+butyrate):propionate ([C2+C4]:C3) ratio in both crossbred cattle and buffalo were not affected by high or low roughage diet, but percentage of acetate and butyrate varied significantly (p<0.05) among treatment groups. Activities of microbial enzymes were comparable among species and different treatment groups. A total number of rumen protozoa were significantly (p<0.05) higher in crossbred cattle than buffaloes along with significantly (p<0.05) higher population in animal fed with high concentrate diet (T_1_).

**Conclusion::**

Rumen microbial population and fermentation depend on constituents of the treatment diet. However, microbial enzyme activity remains similar among species and different treatments. High concentrate diet increases number of rumen protozoa, and the number is higher in crossbred cattle than buffaloes.

## Introduction

The comparison of rumen metabolism of crossbred cattle and buffaloes are quite challenging and interesting to recognize the rumen microbial activities under the same feeding and environmental conditions. The rumen in both cattle and buffaloes is well developed and recognized as fermentation vat to utilize the cellulosic matter and allows the maximal use of fermentation end-products particularly volatile fatty acids (VFAs) and microbial proteins for ruminants [1-4]. Despite these similarities, some differences also exists in terms of feed intake, nutrient digestibility, rumen bacterial and protozoal population, behavioral habits, and their interactions with the environment [1,5,6].

Many *in-vitro* as well as *in-vivo* studies have been carried out concerning digestion, metabolism, rumen microbial population, and physiological conditions in cattle and buffaloes [7-14]. Swamp buffaloes are more efficient than cattle in many aspects, namely, nitrogen (N)-recycling and fiber digestion, ruminal ammonia nitrogen (NH_3_-N) level in relation to efficient fermentation and intake [1,15-17]. High grain diet and or the little addition of soluble carbohydrate results in shift of fermentation pattern, lowered ruminal pH, and few protozoa may be eliminated or inhibited [18].

There is paucity of literature dealing with rearing of cattle and buffalo on similar diets and under same environmental conditions. Therefore, the present study was designed to evaluate the effect of feeding high and low roughage total mixed ration diets on rumen metabolites and enzymatic profiles in crossbred cattle and buffaloes.

## Materials and Methods

### Ethical approval

All the animal experiments were conducted after approval of Institute Animal Ethics Committee of Indian Veterinary Research Institute (IVRI) after approval by the Director IVRI and CPCSEA, Ministry of Environment and Forestry, Government of India.

### Animals, diets, and experimental design

Three crossbred cattle (*Bos taurus×Bos indicus*) and buffaloes (*Bubalus bubalis*) having average body weight of 364±12 and 335±8 kg, respectively, with permanent fistula were selected. Animals were randomly assigned to receive three treatments of 21 days as per 3×3 switch-over design. The concentrate with roughage was fed *ad libitum* in different ratios according to their body weight. The dietary treatments (concentrate mixture, wheat straw, and green fodder) simulated three different feeding systems, viz., (T_1_) 60:20:20, (T_2_) 40:30:30, and (T_3_) 20:40:40 were applied (Table-1). Concentrate mixture consisted crushed maize grain (37%), solvent-extracted soybean meal (20%), wheat bran (40%), mineral mixture (2%), and salt (1%) and analyzed composition of concentrate and roughage have been presented in Table-2.

**Table-1:** Analysed composition (%) of concentrate and roughage.

Attributes	Concentrate mixture (C)	Wheat straw (W)	Green maize (G)
Proximate components			
DM	89.13	91.57	18.97
OM	92.51	92.65	91.21
CP	20.13	2.95	9.22
EE	2.80	0.84	1.01
Cell wall components			
CF	8.35	34.47	38.64
NDF	37.36	74.52	62.97
ADF	10.35	52.84	39.61

DM=Dry matter; OM=Organic matter; CP=Crude protein; EE=Ether extract; CF=Crude fiber; NDF=Neutral detergent fiber; ADF=Acid detergent fiber.

**Table-2:** Ingredient and chemical composition of diets (% on dry matter basis).

Component	TMR-I	TMR-II	TMR-III
		
	60C:20W: 20G	40C:30W: 30G	20C:40W: 40G
Diet ingredients (%)			
Concentrate mixture (C)	60	40	20
Wheat straw (W)	20	30	40
Green maize (G)	20	30	40
Chemical composition			
Organic matter	92.30	92.16	92.05
Crude protein	14.64	11.65	8.89
Ether extract	2.07	1.67	1.30
Neutral detergent fiber	49.59	56.31	62.48
Acid detergent fiber	24.34	32.01	39.06
Total ash	7.69	7.83	7.95

Mineral mixture 2 and common salt 1% as supplement; concentrate mixture=crushed maize, 37%; solvent extracted soybean meal, 20%; wheat bran, 40%; mineral mixture, 2% and salt, 1%. TMR=Total mixed ration

### Sampling and analysis of rumen fluid

During the past two consecutive days of the experiment, rumen fluids (100 ml) were collected into a pre-warmed flask from the fistulated animals at 0, 2, 4, 6 and 8 h post-feeding of each animal. Ruminal digestibility and rumen metabolite concentrations are changed after feeding in a time-dependent manner. Hence, comparison of effect of high and low roughage diet on rumen metabolite and enzymatic profile will also change in due to the course of time post-feeding. Thus, we have considered different time frame to observe the effect of diet based on the mean values of different rumen metabolite and enzymatic parameters. Moreover, digestibility depends on so many factors including interspecies variation. Monitoring different rumen metabolite parameters and enzymatic profile in response to different types of diet, the interspecies variation can be assumed form which the diet and ruminal environment can be manipulated to have a better effect on production status from these animals. Hence, we thought species interaction might add good values to the current research. Immediately, after collection pH of rumen fluid was measured and transported to laboratory for further analysis such as protozoa, VFAs, and other biochemical parameters related to N fractions. Some amounts of content were kept at −20°C for enzymatic study. NH_3_–N was estimated by method described earlier by Weatherburn [19].

### Rumen metabolites

Rumen fluid was separated into two parts; one part was used to determine VFAs which were analyzed using gas chromatograph equipped with a double flame ionization detector [20] and the second part was analyzed for NH_3_N, total N (TN), and trichloroacetic acid precipitable nitrogen (TCA ppt. N) by the standard Kjeldahl procedure [21]. The nonprotein nitrogen (NPN) was calculated as the difference between TN and TCA ppt. N. Lactic acid (LA) concentration in rumen liquor was estimated as per method described by Barker and Summerson [22].

### Enzyme assay

The microbial enzymes from the rumen contents were extracted as per the method described by Hristov *et al*. [23]. For estimation of carboxymethylcellulase (CMCase) and xylanase activity, the reaction mixture (1 ml phosphate buffer [0.1 M, pH 6.8], 0.5 ml enzyme, and 0.5 ml of either CMCase [1.0%] or xylan [0.25%]), were incubated at 39°C for 60 and 30 min, respectively, and the amount of reducing sugars released were estimated [24]. The Avicelase activity was estimated by measuring the amount of reducing sugar (Avicel 1%) released from Avicel using the reaction mixture (1 ml phosphate buffer [0.1 M, pH 6.8], 1 ml enzyme and 0.5 ml of Avicel [1%]) incubated at 39°C for 60 min. Protein and protease estimation was carried out as per the method described by Lowry *et al*. [25], and protease activity was measured using azocasein as substrate [26].

### Ciliate protozoa

The number of protozoa was counted as per the procedure described by Kamra *et al*. [27]. Number of protozoa/ml rumen liquor N=(n×A×D)/(a×v).

### Statistical analysis

The means of all parameters measured were statistically analyzed by analysis of variance procedure and means were statistically compared in rumen fluid at 0, 2, 4, 6, and 8 h post-feeding among different treatment groups (T1, T2 and T3), between both animal species (cattle and buffalo) and among treatment and species (T×S) using the statistical software SPSS (version 20.0). Differences among treatments were analyzed by Duncan’s multiple range test using the generalized linear model of Snedecor and Cochran [28].

## Results

There was non-significant (p>0.05) difference in intake g/kgW^0.75^ and digestibility of dry matter (DM), organic matter (OM), crude protein (CP), ether extract, neutral detergent fiber (NDF), and acid detergent fiber (ADF) in crossbred cattle and buffalo (Table-3). Among treatment, digestibility was influenced in high roughage diet to low roughage diet. In ruminant species, apparent digestibility was increased according to inclusion level of concentrate in diets.

**Table-3:** Feed intakes and apparent digestibility (%) of TMR in cattle and swamp buffaloes receiving the same diets.

Attributes	60C:20W: 20G	40C:30W: 30G	20C:40W: 40G	Mean±SE		SEM	p values		
				
	T1	T2	T3	Cattle	Buffalo		T	S	T×S
DMI, kg/day	6.93^b^±0.34	6.71^ab^±0.34	5.83^a^±0.32	5.91^q^±0.27	7.06^p^±0.25	0.20	0.04*	0.008**	0.91
DMI g/kgW^0.75^	105.49^b^±1.76	101.52^b^±2.77	86.33^a^±2.35	95.79±2.88	99.77±2.51	1.91	0.003**	0.14	0.34
Apparent digestibility, %									
DM	66.61^c^±0.57	62.86^b^±0.82	58.98^a^±0.97	62.96±0.99	62.68±0.99	0.69	0.006**	0.77	0.91
OM	69.90^c^±0.50	67.48^b^±0.68	65.32^a^±0.77	67.58±0.71	67.55±0.69	0.49	0.008**	0.96	0.83
CP	70.55^b^±1.52	64.61^ab^±2.35	62.27^a^±2.51	64.17±2.04	67.45±1.74	1.35	0.03*	0.20	0.73
EE	72.42^b^±1.11	63.49^a^±1.02	62.76^a^±2.04	66.00±1.59	66.45±1.58	1.10	0.004**	0.79	0.71
NDF	49.16^a^±0.68	52.16^a^±1.13	56.68^b^±2.07	51.64±1.39	53.69±1.28	0.95	0.007**	0.22	0.79
ADF	41.06^a^±1.47	44.68^ab^±1.54	47.67^b^±1.43	43.64±1.34	45.30±1.34	0.94	0.01**	0.35	0.94

Mean bearing different superscripts in a column and row differ significantly,

* p<0.05;

** p<0.01. SEM=Standard error of the mean (n=36), T=Dietary treatment, S=Species (crossbred cattle and buffalo), T×S = Interaction between species and dietary treatments, DMI=Dry matter intake, TMR=Total mixed ration, DM=Dry matter, OM=Organic matter, CP=Crude protein, EE=Ether extract, CF=Crude fiber, NDF=Neutral detergent fiber, ADF=Acid detergent fiber, SE=Standard error, C=Concentrate mixture, W=Wheat straw, G=Green maize

### Rumen pH

Feeding of different diet did not alter pH of the rumen fluid significantly (p>0.05) post-feeding among different treatment groups. No significant (p>0.05) variation in ruminal pH was also observed post-treatment between crossbred cattle and buffalo population (Table-4).

**Table-4:** Nitrogen fractions and lactate (mg/dL) in rumen liquor of fistulated crossbred cattle and buffaloes fed various TMR diet.

Attributes	60C:20W: 20G	40C:30W: 30G	20C:40W: 40G	Mean±SE		SEM	p values		
				
	T1	T2	T3	Cattle	Buffalo		T	S	T×S
pH	6.24±0.05	6.26±0.05	6.33±0.07	6.24±0.05	6.31±0.04	0.03	0.587	0.342	0.795
NH_3_-N	11.11^b^±0.66	8.43^a^±0.42	7.36^a^±0.44	8.27^q^±0.53	9.66^p^±0.55	0.39	0.009**	0.023*	0.909
TN	82.83^c^±2.32	65.83^b^±3.81	58.00^a^±3.24	62.00^q^±3.51	75.77^p^±2.80	2.50	0.006**	0.003**	0.257
TCA-ppt. N	58.50^b^±2.54	45.00^a^±2.35	40.33^a^±2.58	47.33±2.76	48.55±2.71	1.91	0.007**	0.684	0.959
NPN	24.33±3.28	20.83±4.00	17.66±2.80	14.66^q^±1.72	27.22^p^±2.86	1.96	0.29	0.005**	0.406
LA	1.76±0.04	1.71±0.04	1.66±0.07	1.74±0.05	1.68±0.04	0.03	0.463	0.396	0.893

Mean bearing different superscripts in a column and row differ significantly,

* p<0.05;

** p<0.01; mg/dL=Milligrams per decilitre, pH=Potential of hydrogen, NH3-N=Ammonia nitrogen, TN=Total nitrogen, TCA-ppt. N=Trichloroacetic acid induced nitrogen precipitation, NPN=Non protein nitrogen, LA=Lactic acid, TMR=Total mixed ration, SEM=Standard error of mean, SE=Standard error, T=Dietary treatment, S=Species (crossbred cattle and buffalo), T×S=Interaction between species and dietary treatments, C=Concentrate mixture, W=Wheat straw, G=Green maize

### Nitrogen fractions

Nitrogen fractions, viz., NH_3_-N, TN, TCA ppt. N and NPN in the rumen liquor in groups (T_1_, T_2_, and T_3_) are presented in Table-4. The concentration of NH_3_-N (mg/dL) was significantly (p<0.01) lower in group T_3_ followed by T_2_ than T_1_. Ruminal NH_3_-N varied significantly (p<0.05) and was found to be higher in buffalo than crossbred cattle. The total nitrogen level (mg/dL) was found to be significantly (p<0.01) higher in buffaloes than crossbred cattle. Among treatments, it was varied significantly (p<0.01) and observed to be highest for T_1_ and lowest for T_3_; however, the value in T_2_ was intermediate between these two groups. The variation in mean values of TCA ppt. N were observed to be non-significant (p>0.05) between crossbred cattle and buffaloes but it varied significantly (p<0.05) within the treatment groups. The NPN value (mg/dl) was significantly (p<0.01) higher for buffaloes than crossbred cattle. Among the treatments groups, NPN value (mg/dL) was non-significant (p>0.05) with each other.

### LA and VFAs

There was no significant (p>0.05) variation observed in LA concentration, neither among the treatment groups nor between crossbred cattle and buffalo population post treatment (Table-4).

The values of total VFAs (TVFAs) and relative percent of acetate, propionate, and butyrate were depicted in Table-5. A significant difference (p<0.05) in TVFAs concentration was observed in the rumen liquor of crossbred cattle and buffaloes. The level of acetate was significantly (p<0.05) higher in crossbred cattle (9.26±0.24) than buffaloes (8.41±0.24). However, no significant (p>0.05) variation in acetate concentration was observed among the treatment groups post-feeding. Whereas propionate concentrations did not differ significantly (p>0.05) among the treatment groups and between cross-bred cattle and buffalo population post-feeding. Butyrate (mmol/dL) concentrations were found to be 2.28±0.17, 1.93±0.09, and 1.65±0.17 for treatments T_1_, T_2_, and T_3_, respectively. The difference was significant (p<0.05) between T_1_ and T_3_ group while T_2_ differed non-significantly (p>0.05) from both of them.

**Table-5:** Effect on TVFA and its fractions (mM/100 ml) and molar proportion (mol/100 mol) in rumen liquor of fistulated crossbred cattle and buffaloes fed various TMR diet.

Attributes	60C:20W: 20G	40C:30W: 30G	20C:40W: 40G	Mean±SE		SEM	p values		
				
	T1	T2	T3	Cattle	Buffalo		T	S	T×S
Acetate, C2	8.95±0.26	9.02±0.39	8.54±0.28	9.26^p^±0.24	8.41^q^±0.24	0.18	0.47	0.02*	0.32
Propionate, C3	2.16±0.12	2.13±0.15	2.00±0.12	2.21±0.12	1.98±0.08	0.07	0.67	0.13	0.77
Butyrate, C4	2.28^b^±0.17	1.93^ab^±0.09	1.65^a^±0.17	2.07±0.13	1.84±0.13	0.09	0.02*	0.20	0.95
TVFA	13.65±0.47	13.36±0.62	12.39±0.50	13.76^p^±0.42	12.50^q^±0.43	0.31	0.22	*0.045	0.64
Acetate, C2	65.77^a^±0.68	67.64^ab^±0.50	69.26^b^±1.15	67.49±0.64	67.62±0.84	0.52	0.02*	0.44	0.45
Propionate, C3	15.77±0.53	15.86±0.46	16.11±0.55	16.02±0.53	15.81±0.26	0.29	0.29	0.24	0.60
Butyrate, C4	16.58^b^±0.89	14.49^ab^±0.41	13.14^a^±1.31	14.99±0.77	14.48±0.89	0.58	0.02*	0.44	0.62
A:P or (C2:C3 ratio)	4.22±0.13	4.30±0.13	4.34±0.13	4.28±0.13	4.29±0.07	0.07	0.32	0.30	0.48
(A+B):P or (C2+C4):C3 ratio	5.29±0.20	5.23±0.17	5.18±0.19	5.25±0.19	5.21±0.09	0.10	0.33	0.21	0.47

Mean bearing different superscripts in a column and row differ significantly,

* p<0.05; TVFA=Total volatile fatty acids, mM/100 ml=Milli-mole per 100 millilitre. C2:C3 ratio=Acetate:propionate ratio, (C2+C4):C3 ratio=(Acetate+butyrate):Propionate ratio. TMR=Total mixed ration, SEM=Standard error of mean, SE=Standard error, T=Dietary treatment, S=Species (crossbred cattle and buffalo), T×S = Interaction between species and dietary treatments, TVFA=Total volatile fatty acids, C=Concentrate mixture, W=Wheat straw, G=Green maize

The molar percent of acetate, propionate, butyrate, A:P ratio and (A+B):P ratio varied non-significantly (p>0.05) between crossbred cattle and buffaloes. Although molar proportion of acetate and butyrate (mol/100 mol) differed significantly (p<0.05) among different treatment groups. Acetate proportion was found to be highest in green fodder rich diet (T_3_) while butyrate proportion was found to be highest in concentrate fodder rich diet (T_1_) (Table-5).

### Rumen enzyme activities

Rumen liquor was collected from all animals and analyzed for rumen enzyme, viz., CMCase, xylanase, avicelase, and protease (Table-6). The result indicated that the microbial enzyme profiles of crossbred cattle and buffaloes were comparable (p>0.05). No significant (p>0.05) variation in rumen microbial enzyme activity was also observed among the treatment group animals post-feeding.

**Table-6:** Effect on enzyme activities (IU/mg protein) in rumen content of fistulated crossbred cattle and buffaloes fed various TMR diet.

Enzyme activity	60C:20W: 20G	40C:30W: 30G	20C:40W: 40G	Mean±SE		SEM	p values		
				
	T1	T2	T3	Cattle	Buffalo		T	S	T×S
CMCase	56.37±3.81	60.78±3.89	68.99±3.46	61.39±3.15	62.70±3.34	2.26	0.07	0.76	0.57
Xylanase	179.55±15.18	200.51±15.85	222.33±11.05	213.43±13.12	188.16±10.27	8.48	0.12	0.13	0.70
Avicelase	27.35±2.93	29.20±2.16	31.79±1.84	31.35±2.31	27.55±1.33	1.35	0.41	0.17	0.55
Protease	162.92±8.33	165.69±7.00	157.66±8.01	158.82±7.28	165.36±5.07	4.41	0.77	0.49	0.97

Unit=nmol of glucose released/min/ml for CMCase and avicelase; nmol of xylose released/min/ml for xylanase; µg hydrolyzed protein/min/ml for protease; The mean values did not differ significantly at level p<0.05. IU/mg=International unit per milligram, CMCase=Carboxymethylcellulase, TMR=Total mixed ration, SEM=Standard error of mean, SE=Standard error, T=Dietary treatment, S=Species (crossbred cattle and buffalo), T×S = Interaction between species and dietary treatments, C=Concentrate mixture, W=Wheat straw, G=Green maize

### Ciliate protozoa

Among the treatment groups, protozoal population of holotrichous and sporotrichosis were found to be significantly higher (p<0.01) in T_1_ and T_2_ than T_3_ group animals, whereas cross-bred cattle population was found to harbor significantly (p<0.01) higher number of both of the protozoa than buffaloes (Table-7). Total protozoa population was significantly (p<0.01) higher in crossbred cattle than buffaloes, but within treatment groups, it was highest for T_1_ and lowest for T_3_ group animals.

**Table-7:** Effect on protozoal population (Log10) of fistulated crossbred cattle and buffaloes fed various TMR diet.

Rumen protozoa	60C: 20W: 20G	40C: 30W: 30G	20C: 40W: 40G	Mean±SE		SEM	p values		
				
	T1	T2	T3	Cattle	Buffalo		T	S	T×S
Holotrichous	4.28^c^±0.04	4.15^b^±0.07	3.96^a^±0.07	4.27^p^±0.03	3.99^q^±0.06	0.04	0.008**	0.006**	0.13
Spirotrichous	5.36^b^±0.04	5.27^a^±0.05	5.23^a^±0.04	5.43^p^±0.02	5.15^q^±0.02	0.03	0.008**	0.009**	0.70
Total protozoa	5.40^c^±0.04	5.31^b^±0.05	5.26^a^±0.04	5.46^p^±0.02	5.18^q^±0.02	0.03	0.008**	0.008**	0.85

Mean bearing different superscripts in a column and row differ significantly. *p<0.05; Log=Logarithm, TMR=Total mixed ration, SEM=Standard error of mean, SE=Standard error, T=Dietary treatment, S=Species (crossbred cattle and buffalo), T×S = Interaction between species and dietary treatments, C=Concentrate mixture, W=Wheat straw, G=Green maize

## Discussion

Feeding of different concentrate roughage ratios was found to have no drastic effect on rumen environment [29]. In crossbred cattle and buffalo, mean value of rumen pH was in agreement with values reported by Franzolin *et al*. [16] and Baraka [30]. A similar type of non-significant (p>0.05) effect on diurnal ruminal pH was also documented by Chanthakhoun *et al*. [1] between swamp buffalo and beef cattle fed on rice straw.

There was no significant variation in protease activity between buffalo and cattle, but from the enzyme table, it is evident that protease activity is higher in buffaloes (165.36±5.07 IU/mg protein) than cattle (158.82±7.28 IU/mg protein). The difference may not be significant but its effect may be enough leading to higher NH3-N concentration in buffaloes. Ruminal NH_3_-N varied significantly (p<0.05) and was found to be higher in buffalo than crossbred cattle which was in agreement with Khajarern and Khajarern [31]. The higher concentration of NH_3_-N in buffaloes indicates higher proteolytic activity in the rumen of buffaloes than crossbred cattle although the variation in enzyme activity was not significant [32]. Suwanlee and Wanapat [33] reported that when ruminal NH_3_-N increased from 1.7 to 5.6 mg%, total bacterial count, digestibility of DM, NDF and ADF were increased. In the current study, it is evident from Tables-3 and 4 that the overall trend suggests an increase in ruminal NH3-N level occurs along with increase in DM intake (DMI) among different feeding groups, although the level of variation in terms of significance was not found exactly similar. In case of interspecies difference, buffaloes have higher ruminal NH3-N than cattle along with higher DMI, although the difference in DMI was non-significant. We have removed protozoal population from this context in the revised manuscript as protozoal population is largely dependent on species of animal, type, and source of diet which we have mentioned in the later part of the manuscript. Increased level of ruminal NH_3_-N resulted from increased DMI [29]. However, Chanthakhoun *et al*. [1] reported that NH3-N concentration of buffalo did not differ from cattle although nutrient digestibilities particularly those of DM, OM, CP, NDF, and ADF was significantly (p<0.05) higher in buffalo than cattle.

The values of TN, TCA ppt. N (mg %) and LA (mg/ml) in the rumen liquor increased significantly (p<0.05) with the increase in the proportion of concentrate mixture in the diet of animals. This could be due to increased soluble carbohydrates and proteins in high concentrate diet. The increased nitrogen fractions might be due to increase in the nitrogen intake by the animals with increasing proportion of concentrate in the diet. Higher concentration of ammonia reflecting better activity of intracellular deaminases and salivary recycling of urea [34] and maintenance of nitrogen balance positive due to the greater efficiency of utilization of NH_3_N by ruminal bacteria [35].

Wanapat and Pimpa [29] reported that LA (mg/ml) in the rumen liquor increased significantly (p<0.05) with the increase in the proportion of concentrate mixture in the diet of animals; however, in the present study, there was no such effect observed on LA concentration between and within the treatment groups.

Significantly (p<0.05) higher TVFAs concentration was observed in the rumen liquor of crossbred cattle than buffaloes in the current study which was in similar trend with Chanthakhoun *et al*. [1] and Franzolin *et al*. [16] where higher VFA concentration was observed in cattle than buffaloes although the difference was non-significant, while Cutrignelli *et al*. [36] reported that buffaloes produced higher rumen VFAs than cattle. Although several factors such as anatomy and physiology of digestive system, feed intake and digestibility, rumen microbial metabolism, and rumen ciliate protozoal population [37,38] may be involved for this interspecies variation in VFA production but the exact metabolic mechanism is not known [39]. However, it has been observed that the pH and ammonia concentration of rumen liquor decreased while production of TVFA increased in the absence of rumen ciliate protozoa in lambs [40]. TVFAs were found at normal concentrations of 70-130 mmol/L and also proportions of acetate, propionate, and butyrate in this study were in accordance with Hungate [41]. Among treatments, variation in the level of TVFA was non-significant (p>0.05). The proportion of VFAs (acetate, propionate, and butyrate) was not affected by energy sources which were in agreement with Hoover *et al*. [42].

Franzolin *et al*. [16] described buffaloes had lower production of acetic acid than cattle (58.7 vs. 61.6 mol/100 mol) and higher proportion of propionic acid (27.4 vs. 23.6 mol/100 mol). There was no difference in the butyric acid production between the buffaloes (13.6 mol/100 mol) and cattle (14.8 mol/100 mol).

Enzyme activities in terms of CMCase, xylanase, avicelase, and protease activity were found to be similar in rumen content of cattle and buffalo. The probable reason of similar enzyme activity in these two species could be due to the same microbial population in the rumen fed with similar type of diets. Some workers reported higher cellulose digestibility in buffaloes than cattle [43], whereas others did not observe any difference [44].

Total protozoa population was significantly (p<0.01) higher in crossbred cattle than buffaloes but within treatment groups and it was highest for concentrate rich diet (T_1_) and lowest for low concentrate diet (T_3_) group animals. It was in agreement with Franzolin *et al*. [16] who reported similar type of interspecies difference in total rumen protozoal count. They also reported that protozoal count and type may vary according to the type and source of diet. Other previous reports also depicted that zebu cattle had higher numbers of rumen protozoa than the buffaloes [1,45]; however inversely, it was higher in buffalo than in cattle rumen [3], but no differences were reported by Kurar *et al*. [46].

## Conclusion

From the present study, it may be concluded that optimum roughage to concentrate is one of the dietary means which influence microbial population for rumen fermentation depending on constituents of the treatment diet. Activities of microbial enzymes were comparable among species and different treatment groups. A total number of rumen protozoa were higher in crossbred cattle than buffaloes along with higher in animal fed high concentrate diet. Further, comparison of the rumen metabolism in crossbred cattle and buffaloes could be of scientific interest to improve the rumen microbial activities of these species by manipulating ruminal microenvironment for better digestibility and utilization of the similar feedstuffs.

## Authors’ Contributions

SKS processed and analyzed the samples. VBC, PS, and LCC designed the study and corrected the manuscript. SKS, MG, and SS analyzed the data and prepared the manuscript. All authors read and approved the final manuscript.

## References

[1] Chanthakhoun V, Wanapat M, Kongmun P, Cherdthong A (2012). Comparison of ruminal fermentation characteristics and microbial population in swamp buffalo and cattle. Livest. Sci.

[2] Lwin K.O, Kondo M, Ban-Tokuda T, Lapitan R.M, Del-Barrio A.N, Fujihara T, Matsui H (2012). Ruminal fermentation and microbial ecology of buffaloes and cattle fed the same diet. Anim. Sci. J.

[3] Jabari S, Eslami M, Chaji M, Mohammadabadi T, Bojarpour M (2014). Comparison digestibility and protozoa population of Khuzestan water buffalo and Holstein cow. Vet. Res. Forum.

[4] Franzolin R, Wright A.G (2016). Microorganisms in the rumen and reticulum of buffalo (*Bubalus bubalis*) fed two different feeding systems. BMC Res. Notes.

[5] Khejornsart P, Wanapat M, Rowlinson P (2011). Diversity of anaerobic fungi and rumen fermentation characteristic in swamp buffalo and beef cattle fed on different diets. Livest. Sci.

[6] Rafiei M, Chaji M, Mohammadabadi T, Sari S (2013). The comparison digestibility of steam treated sugarcane pith by rumen bacteria or rumen microorganisms of Holstein cow and buffalo of Khuzestan. J. Rumin. Res.

[7] Vinh N.T, Wanapat M, Khejornsart P, Kongmun P (2011). Studies of diversity of rumen microorganisms and fermentation in swamp buffalo fed different diets. J. Anim. Vet. Adv.

[8] Wright A.D.G, Klieve A.V (2011). 2011 the complexity of the rumen microbial ecology preclude methane mitigation?. Anim. Feed. Sci. Technol.

[9] Chaji M, Mohammadabadi T (2011). The investigation of *in vitro* fermentation of sugarcane pith treated with low temperature steam and sulfuric acid by isolated rumen microbial fractions. Anim. Nutr. Feed. Technol.

[10] Franzolin R, St-Pierre B, Northwood K, Wright A.D.G (2012). Analysis of rumen methanogen diversity in water buffaloes (*Bubalus bubalis*) under three different diets. Microb. Ecol.

[11] Wanapat M, Pilajun R, Polyorach S, Cherdthong A, Khejornsart P, Rowlinson P (2013). Effect of carbohydrate source and cottonseed meal level in the concentrate on feed intake, nutrient digestibility, rumen fermentation and microbial protein synthesis in swamp buffaloes. Asian Aust. J. Anim. Sci.

[12] Nathani N.M, Patel A.K, Dhamannapatil P.S, Kothari R.K, Singh K.M, Joshi C.G (2013). Comparative evaluation of rumen metagenome community using qPCR and MG-RAST. AMB Expr.

[13] Shakarami F, Chaji M, Eslami M, Mohammadabadi T, Bojarpour M (2015). The comparison of *in vitro* digestibility of wheat straw by rumen anaerobic fungi of Khuzestan buffalo and Holstein cattle. Iran J Apppl. Anim. Sci.

[14] Wadhwa M, Bakshi M.P.S, Makkar H.P.S (2016). Modifying gut microbiomes in large ruminants:Opportunities in non-intensive husbandry systems. Anim. Front.

[15] Wanapat M, Sommart K, Wachirapakorn C, Uriyapongson S, Wattanachant C, Wanapat M, Sommart K (1994). 1994 advances in swamp buffalo nutrition and feeding. Proceeding of the 1^st^Asian Buffalo Association Congress.

[16] Franzolin R, Rosales F.P, Soares W.V.B (2010). Effects of dietary energy and nitrogen supplements on rumen fermentation and protozoa population in buffalo and zebu cattle. Rev. Bras. Zootec.

[17] Wanapat M, Phesatcha K, Kang S (2016). Rumen adaptation of swamp buffaloes (*Bubalus bubalis*) by high level of urea supplementation when fed on rice straw-based diet. Trop. Anim. Health Prod.

[18] van Soest P.J (1994). 1994 Ecology of the Ruminant.

[19] Weatherburn M.W (1967). Phenol hypochlorite reaction for determination of ammonia. Anal. Chem.

[20] Cottyn B.G, Boucque C.V (1968). Rapid method for the gas chromatographic determination of volatile fatty acids in rumen fluid. J. Agric. Food Chem.

[21] AOAC (1995). 1995 of Official Analytical Chemist.

[22] Barker S.B, Summerson W.H (1941). The calorimetric determination of lactic acid in biological materials. J. Biol. Chem.

[23] Hristov A.N, McAllister T.A, Cheng K.J (1999). Effect of diet, digesta processing, freezing and extraction procedure on some polysaccharide degrading activities of ruminal contents. Can. J. Anim. Sci.

[24] Miller G.L (1959). Modified DNS method for reducing sugars. Anal. Chem.

[25] Lowry O.H, Rosenbrough N.J, Farr A.L, Randall R.C (1951). Protein measurement with the folin-phenol reagent. J. Biol. Chem.

[26] Brock F.M, Forsberg C.L, Buchanan-Smith J.G (1982). Proteolytic activity of rumen microorganisms and effects of proteinase inhibitors. Appl. Environ. Microbiol.

[27] Kamra D.N, Sawal R.K, Pathak N.N, Kewalramani N, Agarwal N (1991). Diurnal variation in ciliate protozoa in the rumen of black buck (*Antilope cervicapra*) fed green forage. J. Appl. Microbiol.

[28] Snedecor G.W, Cochran W.G (1994). 1994 Methods.

[29] Wanapat M, Pimpa O (1999). Effect of ruminal NH_3_-N levels on ruminal fermentation, purine derivatives, digestibility and rice straw intake in swamp buffaloes. Asian Australas. J. Anim. Sci.

[30] Baraka T.A (2012). Comparative studies of rumen pH, total protozoa count, generic and species composition of ciliates in camel, buffalo, cattle, sheep and goat in Egypt. J. Am. Sci.

[31] Khajarern S, Khajarern J.M (1990). Feeding swamp buffalo for milk production. Feeding dairy cows in the tropics. FAO Anim. Prod. Health Pap.

[32] Bhatia S.K, Pradhan K, Sangwan D.C, Singh S, Sagar V (1995). Ruminal degradation of fibrous component of various feeds in cattle and buffalo. Indian J. Anim. Sci.

[33] Suwanlee S, Wanapat M, Wanapat M, Sommart K (1994). 1994 of ruminal NH_3_-N on total volatile fatty acid, bacterial population and digestibility in swamp buffaloes. Proceedings of the 1^st^ Asian Buffalo Association Congress.

[34] Tewatia B.S, Bhatia S.K (1998). Comparative ruminal biochemical and digestion related physiological characteristics in buffaloes and cattle fed a fibrous diet. Buffalo J.

[35] Gandra J.R, Freitas J.E, Barletta R.V, Filho M.M, Gimenes L.U, Vilela F.G, Baruselli P.S, Rennó F.P (2011). Productive performance, nutrient digestion and metabolism of Holstein (*Bos taurus*) and Nellore (*Bos taurus indicus*) cattle and Mediterranean Buffaloes (*Bubalis bubalis*) fed with corn-silage based diets. Livest. Sci.

[36] Cutrignelli M.I, D’urso S, Tudisco R, Grossi M, Piccolo V (2007). Effect of ruminant species (*Bovine* vs buffalo) and source of inoculum (rumen liquor vs faeces) on *in vitro* fermentation. Ital. J. Anim. Sci.

[37] Franzolin R (1994). Feed efficiency:A comparison between cattle and buffalo. Buffalo J. Suppl.

[38] Calabro S, Moniello G, Piccolo V, Bovera F, Infascelli F, Tudisco R, Cutrignelli M.I (2008). Rumen fermentation and degradability in buffalo and cattle using the *in vitro* gas production technique. J Anim. Physiol. Anim. Nutr.

[39] Calabrò S, Williams B.A, Piccolo V, Infascelli F, Tamminga S (2004). A comparison between buffalo (*Bubalus bubalis*) and cow (*Bos taurus*) rumen fluids in terms of the *in vitro* fermentation characteristics of three fibrous feedstuffs. J. Sci. Food Agric.

[40] Santra A, Karim S.A (2002). Influence of ciliate protozoa on biochemical changes and hydrolytic enzyme profile in the rumen ecosystem. J. Appl. Microbiol.

[41] Hungate R.E (1966). 1966 Rumen and its Microbes.

[42] Hoover W.H, Tucker C, Harris J, Sniffen C.J, de Ondarza M.B (2006). Effects of non-structural carbohydrate level and starch:Sugar ratio on microbial metabolism in continuous culture of rumen contents. Anim. Feed. Sci. Technol.

[43] Ichinohe T, Orden E.A, Delbarrio A.N, Lapitan R.M, Fujihara T, Cruz L.C, Kanai Y (2004). Comparison of voluntary feed intake, rumen passage and degradation kinetics between crossbred Brahmam cattle (*Bos indicus*) and swamp buffaloes (*Bubalus bubalis*) fed a fattening diet based on corn silage. J. Anim. Sci.

[44] Lapitan R.M, Del-Barrio A.N, Katsube O, Ban-Tokuda T, Orden E.A, Robles A.Y, Kanai L.C, Cruz Y, Fujihara T (2008). Comparison of fattening performance in Brahman grade cattle (*Bos indicus*) and crossbred water buffalo (*Bubalus bubalis*) fed on high roughage diet. J. Anim. Sci.

[45] Dehority B.A (2003). 2003 Microbiology.

[46] Kurar C.K, Gupta B.N, Mohini M (1988). Protozoal status in strained rumen liquor of cattle and buffaloes. Indian J. Anim. Sci.

